# DNA甲基化在肺癌早期诊断中的研究进展

**DOI:** 10.3779/j.issn.1009-3419.2012.04.08

**Published:** 2012-04-20

**Authors:** 小明 邱, 艳洁 乔, 斌 刘, 洋 李

**Affiliations:** 300052 天津，天津市肺癌研究所，天津市肺癌转移与肿瘤微环境重点实验室，天津医科大学总医院肺部肿瘤外科 Tianjin Key Laboratory of Lung Cancer Metastasis and Tumor Microenvironment, Tianjin Lung Cancer Institute, Department of Lung Cancer Surgery, Tianjin Medical University General Hospital, Tianjin 300052, China

**Keywords:** 肺肿瘤, DNA甲基化, 早期诊断, Lung neoplasms, DNA methylation, Early diagnosis

## Abstract

肺癌是当今世界上对人类健康和生命威胁最大的恶性肿瘤，目前缺乏理想的早期诊断手段。在肺癌发生早期就存在很多DNA甲基化的改变，检测DNA甲基化有望成为肺癌早期诊断的重要手段。

## Abstract (en)

Lung cancer is the leading cause of cancer-related death and thus a major health problem nowadays. No early diagnostic method is ideal up to now. Changes in DNA methylation occur on early stage of lung cancer. Detection of DNA methylation is expected to be an important method in early diagosis of lung cancer.

肺癌是当今世界上对人类健康和生命威胁最大的恶性肿瘤，也是临床治疗疗效最差的恶性肿瘤。据估计2008年全世界有160万人被诊断为肺癌，占所有肿瘤的13%，约140万人死于肺癌，占所有肿瘤死亡的18%^[1]^。2010年在美国约有16万人死于肺癌，在男性和女性均位列首位。其中，男性因肺癌死亡8.6万人，占所有肿瘤死亡的29%，超过了位列第2-4位的前列腺癌、结直肠癌和胰腺癌死亡人数的总和；女性肺癌死亡7.1万人，占所有肿瘤死亡的26%，超过了位于第2、3位的乳腺癌和结直肠癌死亡人数的总和^[2]^。肺癌总的5年生存率仅为16%，远远低于男性发病首位的前列腺癌(100%)和女性发病首位的乳腺癌(90%)。肺癌如果在局限期得到发现，则其5年生存率能提高到53%，然而目前仅有15%的患者能在早期发现。

与前列腺癌和乳腺癌相比，肺癌目前缺乏理想的早期筛查手段。胸片和痰细胞学检测已用于肺癌的筛查，具有经济和方便的优势，但其敏感性和特异性不高，而且在高危人群进行筛查并不能降低肺癌的死亡率^[3, 4]^。低剂量螺旋CT有很高的敏感性，能发现肺内数毫米的微小病变，新筛查出的肺癌病例80%以上为Ⅰ期^[5-7]^，如果立即手术其10年生存率达92%，而所有未治疗的Ⅰ期患者均在5年内死亡^[8]^。然而应用低剂量螺旋CT进行肺癌筛查也存在一些问题：首先作为大规模筛查手段其费用较高；其次敏感性很高，能检查出大量非钙化结节，其中非肿瘤性结节远远高于肿瘤性结节，特异性不高。有研究^[9]^显示低剂量螺旋CT发现的非钙化结节中实际为肿瘤的结节不到2%，而确定这些结节的良恶性需要定期的CT随访检查、活检或者手术，对大部分良性结节患者这将造成巨大的精神负担、经济负担甚至身体创伤。

由于影像学技术在肺癌早期筛查中未能显示出良好的效果，人们开始将目光转向肺癌早期诊断的分子标志物，遗憾的是迄今为止没有发现一个敏感性和特异性较高的分子标志物。近年来肺癌表观遗传学研究进展特别是DNA甲基化的研究^[10]^发现，肺癌发生早期就存在很多特有的肿瘤相关基因(其中包括癌基因和肿瘤抑制基因)甲基化程度的改变，为肺癌的早期诊断标志物的探索提供了新的契机。

## 1 DNA甲基化的原理及应用

DNA甲基化是指在甲基转移酶的催化下，DNA中CG两个核苷酸的胞嘧啶被选择性地添加甲基基团的化学修饰现象^[11]^。DNA甲基化通常发生在基因的5’端启动子和第1外显子"CpG岛"区域，长约1 kb，能够引起染色质结构、DNA构象、DNA稳定性及DNA与蛋白质相互作用方式的改变，从而抑制基因转录和表达^[12, 13]^。近来研究^[14]^发现DNA甲基化还发生在基因转录起始点上游2 kb左右称为"CpG岛岸"(CpG island shore)的区域，绝大部分组织特异性的DNA甲基化发生在"CpG岛岸"而非"CpG岛"区域，在结肠癌中大部分肿瘤特异的DNA甲基化差异区域分布在"CpG岛岸"。由于DNA甲基化出现在几乎所有肿瘤中，并且发生在癌前病变和癌变早期，因此是肿瘤早期诊断的理想标志物^[15, 16]^。DNA甲基化检测的优势在于，通过PCR扩增可以检测很微量的组织DNA，具有很高的敏感性，并且DNA发生甲基化的区域明确，便于设计引物或探针检测。某些基因的甲基化还可以在体液中检测到，因此非常适合无创检测应用于肿瘤的早期诊断^[17, 18]^。然而近年来通过高通量的芯片检测发现每个基因都有一个以上的甲基化位点，任何一个位点的甲基化都没有出现在所有肿瘤中，某一位点的检测只能诊断出一个亚类的肿瘤，而且不同人的甲基化模式也不相同，因此对于基因的表达和肿瘤的发生起到关键作用的甲基化位点和模式还需要进一步的研究^[14, 19-21]^。近年来已有大量DNA甲基化的研究^[20-22]^，特别是肿瘤中单个或多个基因的甲基化的检测，结合分析其在疾病发生发展中的作用以及与临床病理特征的关系，可以用于疾病的早期筛查、预防、诊断治疗与预后分析。

## 2 DNA甲基化在肺癌早期诊断中的应用

DNA甲基化改变常发生于肿瘤形成过程，包括全基因组水平DNA低甲基化和CpG岛高甲基化^[23]^。肿瘤组织中全基因组水平DNA低甲基化可以激活原来保持沉默的原癌基因表达，而高甲基化使DNA转录受到抑制，使经典抑癌基因、细胞周期调控基因和DNA错配修复基因不表达或低表达从而引发肿瘤^[24-27]^。因此检测这些基因是否有高甲基化将有望在肺癌发生的早期发现肿瘤。对于DNA甲基化在肺癌早期诊断中的应用探索，也经历了从单基因检测到多基因联合检测、从肺癌组织中检测到各种无创微创介质检测的发展过程。

### 2.1 肺癌组织DNA甲基化的检测

DNA甲基化标志物要作为早期诊断的指标，首先必须证实其与肺癌的发生发展有关，在肺癌组织中有高甲基化，并且最好能在早期肺癌甚至原位癌或者癌前病变中就能检测到。而肿瘤的发生是多步骤、多因素并涉及很多基因改变的复杂过程，其中抑癌基因的失活是肿瘤发生的重要分子基础，早期的研究集中验证了各个已知的抑癌基因在肺癌组织中的甲基化和基因表达情况，并分析其与肿瘤类型、分期等的关系，以期作为早期筛查的标志物。

抑癌基因*p16*是重要的细胞周期调控基因，能负责调节细胞的增殖及分裂，一旦失活则会导致细胞恶性增殖发生恶性肿瘤。Belinsky等^[28]^用甲基化特异性PCR(methylation specific PCR, MSP)的方法检测了18例肺鳞癌患者，发现11例(61.1%)患者的p16启动子发生了较高水平的甲基化，并且在其中75%的癌旁原位癌组织中检测到p16启动子甲基化，甚至在很早期癌前病变基底细胞增生(17%)和鳞状上皮化生(24%)中就发现p16启动子甲基化，因此被认为可作为肺癌早期诊断的标志物。而另外的报道有不同的结果，Kim等^[29]^检测了185例肺癌组织，其中仅50例(27%)发现p16启动子甲基化，其中鳞癌(41%)的甲基化阳性率高于腺癌(22%)。不同文献^[30-42]^报道的p16甲基化的阳性率差异较大(21.9%-80.2%)，其差异可能是由于检测的方法、选择的人群、肺癌的病理类型及检测样本量的不同引起。

抑癌基因*APC*通过调节微管的稳定性，影响细胞迁移能力，起到维持染色体稳定性的作用，其失活与结肠癌等多种肿瘤的发生有关。Brabender等^[43]^报道，在91例非小细胞肺癌(non-small cell lung cancer, NSCLC)患者的肿瘤组织中，86例(95%)检测到APC启动子区甲基化阳性，而且这些患者的正常肺组织中也检测到80例(88%)阳性，而在10例正常对照组人群肺组织标本中仅2例(20%)阳性，差异明显，有望成为诊断标志物。Usadel等^[44]^也报道了在99例原发性肺癌组织中发现95例(96%)有APC启动子甲基化。而大部分其它研究报道的阳性率则不高，Kim等^[45]^检测了99例外科切除肺癌组织，其中仅48例(48%)发现APC启动子甲基化。而Lin等^[46]^检测了123例Ⅰ期NSCLC，其中仅49例(40%)检测到APC启动子区域甲基化。Virmani等^[47]^用MSP的方法检测发现106例NSCLC组织和细胞系中56例(53%)甲基化，50例小细胞肺癌中13例(26 %)甲基化，而68例非癌组织中3例(4%)甲基化。Zhang等^[48]^检测了78对NSCLC组织和配对的癌旁组织中APC基因甲基化的情况，其中肺癌中甲基化率为56%(44例)，而癌旁组织中仅为13%(10例)，尽管阳性率不高，但是在正常组织和癌组织中差异明显，仍有可能成为早期诊断的标志物。

*RASSF1A*作为一个肿瘤抑制基因，能够通过稳定微管、调控细胞周期和诱导凋亡而发挥抑制肿瘤发生的作用^[49]^。Dammann等^[50]^从3号染色体短臂上克隆并证实RASSF1A基因在肺癌中表达缺失，并且主要与启动子区域高甲基化有关。Endoh等^[51]^检测了100例NSCLC组织，发现其中42例(42%)有RASSF1A甲基化，但其结果提示*RASSF1A*甲基化与肿瘤的类型、分期等没有相关性。但Tomizawa等^[52]^检测110例Ⅰ期肺腺癌发现35例(32%)有*RASSF1A*甲基化，并且与胸膜、血管侵犯和分化差相关。Zhang等^[48]^检测78例NSCLC组织和配对的癌旁组织中*RASSF1A*基因甲基化的情况，其中肺癌中甲基化率为39.7%(31例)，而癌旁组织中仅为7.7%(6例)。其它报道^[53-57]^的肺癌中*RASSF1A*基因甲基化率在29%-41%。

其它研究较多的抑癌基因和肿瘤相关基因包括*DAPK*、*FHIT*、*DLEC1*、*RARβ*、*hMLH1*、*MGMT*、*CDH1*、*CDH13*、*CADM1*、*CALCA*等^[58]^，经过这些研究大量基因被证实在肺癌组织中存在不同程度的甲基化，其差异可能源于采取的检测方式、检测的人群以及病理类型的不同，但总的来说单个基因甲基化的阳性率不高，无法达到肺癌早期筛查的标准。而且大多数研究仅仅检测肿瘤组织的甲基化情况，缺乏对照，无法评价其作为诊断指标的敏感性和特异性。因此进一步的研究还需要探索和发现敏感性和特异性更高的DNA甲基化标志物，或者联合检测多个DNA甲基化标志物，以提高其作为早期诊断指标的敏感性和特异性。

随着研究的深入和技术的进步，近年来DNA甲基化的研究则侧重于运用高通量检测技术以验证和评价多个已知的甲基化位点作为肺癌早期诊断的价值，并探索和发现新的肺癌甲基化标志物。甲基化荧光定量PCR(MethyLight)是一种荧光实时定量甲基化特异性PCR技术，通过引物和探针设计，可以同时检测多个目的基因的甲基化情况。Feng等^[59]^应用MehtyLight技术检测了49对NSCLC组织和癌旁组织的27个基因甲基化情况，发现联合检测*RASSF1*、*DAPK1*、*BVES*、*CDH13*、*MGMT*、*KCNH5*、*RARβ*和*CDH1*的甲基化情况在肺癌组织中阳性率为80%，而在癌旁组织中仅为14%。Jin等^[60]^检测了72例肺癌患者肿瘤和癌旁组织的*p16*、*APC*、*CDH13*、*RARβ*、*RASSF1*、*RUNX3*和*MYOD1* 7个基因甲基化情况，发现鳞癌和腺癌的甲基化模式有差异。Tsou等^[61]^应用MehtyLight技术检测了51例肺腺癌，38例肺癌患者的远癌肺组织和11例非肺癌患者的肺组织的28个甲基化位点，其中*CDH13*、*CDKN2A EX2*、*CDX2*、*HOXA1*、*OPCML*、*RASSF1*、*SFPR1*和*TWIST1*在腺癌中高甲基化，而联合*CDKN2A EX2*、*CDX2*、*HOXA*1和*OPCML*用于检测肺腺癌和癌旁组织的敏感性达到94%，特异性达到90%。Anglim等^[62]^应用MehtyLight技术检测了45对鳞癌和癌旁组织的42个甲基化位点，发现鳞癌中*GDNF*、*MTHFR*、*OPCML*、*TNFRSF25*、*TCF21*、*PAX8*、*PTPRN2*和*PITX2*这8个基因甲基化程度明显高于癌旁组织，联合这8个基因的甲基化对于检测鳞癌达到95.6%的敏感性和特异性。甲基化发生在肿瘤发生的非常早期，Selamat等^[63]^分析了249例组织样本的15个甲基化位点，包括正常的癌旁组织、非典型腺瘤样增生(atypical adcnomatous hyperplasia, AAH)组织、原位腺癌(adenocarcinoma insitu, AIS)和侵袭性腺癌组织，结果发现*CDKN2A EX2*和*PTPRN2*的甲基化程度在AAH时已经明显升高，而*2C35*、*EYA4*、*HOXA1*、*HOXA11*、*NEUROD1*、*NEUROD2*和*TMEFF2*的甲基化程度在AIS时明显升高，而*CDH13*、*CDX2*、*OPCML*、*RASSF1*、*SFRP1*及*TWIST1*的高甲基化和全基因组低甲基化主要出现在侵袭性腺癌中，这些结果将有助于癌前病变和早期肺癌的诊断。

Ehrich等^[64]^利用以质谱分析为基础的胞嘧啶甲基化谱分析(cytosine methylation profiles)技术分析了96例患者肺癌和癌旁组织的47个甲基化位点，发现*CLEC3B*、*MGP*、*RASSF1*、*SDK2*、*SERPINB5*和*XAGE1A*这6个基因的甲基化在肺癌组织中明显高于相应癌旁正常肺组织。限制性标记基因组扫描(restriction landmark genomic scanning)通过二维电泳在一块胶上最多可以分析2, 000个启动子序列，运用该技术Dai等^[65]^对1, 184个CpG岛的研究发现11个基因和6个ESTs在肺癌和正常组织中甲基化有差异，其中*GNAL*和*PDX1*基因的甲基化比例超过50%。

芯片技术的发展为DNA甲基化研究提供了更好的平台，具有高通量、大规模、高灵敏性等特点。甲基化芯片能将所有已知的甲基化位点集成在一张芯片上，同时检测整个肿瘤甲基化的变化，从而更深入地理解肺癌发生发展中DNA甲基化的模式，筛选出在肺癌中有诊断价值的新的DNA甲基化标志物^[66, 67]^。Field等^[68]^运用甲基化芯片技术设计检测59个候选基因的245个甲基化位点，发现鳞癌中*ADPRH*、*GP1BB*、*RARβ*和*TMEFF2*高甲基化，腺癌中*CDKN1C*、*MGMT*和*TMEFF2*高甲基化。Fukasawa等^[69]^运用类似方法研究分析288个肿瘤相关基因的启动子区域，发现28个潜在的甲基化标志物，进一步研究发现*PAX3*和*ASC*在肺癌组织中有很高的甲基化率。Rauch等^[70, 71]^使用含有12, 192个CpG岛的甲基化芯片，发现包括肺癌中包括*DLEC1*和*PAX7*等基因在内的50个甲基化位点有明显差异，进一步研究发现*HOXA9*和*HOXA7*等基因可作为鳞癌诊断的标志物。Bibikova等^[72]^应用基于磁珠的液相甲基化芯片技术在腺癌中对807个基因的1, 505个CpG位点进行分析，发现一组特异性的甲基化谱包括*ASCL2*、*CDH13*、*HOXA11*、*HOXA5*、*NPY*、*RUNX3*、*TERT*和*TP73*。目前最新的甲基化芯片包括了近3万个甲基化位点，能覆盖了整个基因组范围95%以上的甲基化位点。

基因表达谱芯片技术也被用于探索潜在的甲基化位点，通过基因表达谱芯片对比甲基化抑制剂5-Aza-dC处理前后基因表达谱的变化，可以筛选出大量甲基化基因。Shames等^[73]^通过这种方法发现132个肿瘤特异性的甲基化位点，其中7个(*ALDH1A3*、*BNC1*、*CCNA1*、*CTSZ*、*LOX*、*MSX1*和*NRCAM*)在肺癌和癌旁组织中差异明显，可作为肺癌诊断标志物。

通过这些高通量的检测方法可以迅速地筛选出很多潜在的甲基化标记物，但是这些新的标记物必须通过传统方法在原发肿瘤组织中进行验证。目前不同文献报道的甲基化位点各不相同，很多基因并没有重复性，因此还需要更多的试验进行探索和验证，并尽可能地排除人种、肿瘤病理类型和数据分析方法等因素的影响。

DNA甲基化作为肺癌早期诊断的分子标志物，其敏感性和特异性是研究关注的焦点。在以往的研究中DNA甲基化对肺癌诊断的敏感性和特异性也存在很大的差异，目前尚未发现敏感性和特异性非常高的DNA甲基化标志物。在不断探索和筛选敏感性和特异性更高的DNA甲基化标志物的同时，人们也在不断优化组合现有的DNA甲基化标志物，通过多基因联合检测以提高早期诊断的敏感性和特异性。Shivapurkar等^[74]^报道了HS3ST2、DAPK和TNFRSF10C组合区分肺癌和癌旁组织的受试者工作特征曲线的曲线下面积达到0.959，用于诊断肺癌有很高的敏感性和特异性。Ehrich等^[64]^报道的6个基因的组合分辨肺癌和癌旁组织的敏感性和特异性均大于95%。而Bibikova等^[72]^报道的55个基因的组合区分腺癌和癌旁组织的敏感性达到100%，特异性达到92%。Tsou等^[61]^报道了5个基因的组合诊断肺腺癌的敏感性达94%，特异性达90%。Anglim等^[62]^报道了8个基因组合检测鳞癌达到95.6%的敏感性和特异性。Zhang等^[48]^报道了5个基因的组合用于诊断肺癌的敏感性达到83.64%，特异性达74.0%。尽管这些报道非常鼓舞人心，但是这些结果还需要更大量的人群进行验证。同时，肺癌组织的检测并不能用于肺癌的早期筛查中，因此这些标志物还需要在早期筛查中常用的无创微创介质如血液、痰液中进行验证，以评价其敏感性和特异性。

### 2.2 血液中DNA甲基化的检测

外周血是肺癌早期筛查和诊断比较理想的样本，肿瘤患者血液中存在肿瘤细胞释放的高水平的循环DNA，并且与原发肿瘤有着相同的基因改变^[75, 76]^。Usadel等^[44]^在47%的肺癌患者血清中发现APC基因甲基化。Ulivi等^[40]^报道肺癌患者血清中*p16*和CDH13的甲基化阳性率分别为26%和23%。Liu等^[41]^报道肺癌患者血清中*p16*甲基化为72%，Bearzatto等^[77]^报道肺癌患者血清中*p16*甲基化为55%。Wang等^[78]^也报道肺癌患者血清中*RASSF1A*甲基化为33.8%(27/80)，而在良性肺疾病患者和正常人血清中没有检测到。Kneip等^[79]^报道了*SHOX2*甲基化诊断肺癌的敏感性为60%，特异性达到90%，且分期越晚，敏感性越高。

同样也有大量研究联合检测多个甲基化标志物，以提高诊断的敏感性和特异性。Esteller等^[76]^检测了22例肺癌患者的肿瘤组织和血清中DNA的*p16*、*DAPK*、*GSTP1*和*MGMT*的甲基化情况，结果显示15例(68%)的患者至少有1个基因甲基化，而在这些患者血液中11例(72%)患者发现基因甲基化。Fujiwara等^[80]^检测了91例肺癌患者，发现血清中*MGMT*甲基化为18.7%，*p16*为15.4%，*RASSF1A*为12.1%，*DAPK*为11.0%，*RARβ*为6.6%；以这5个基因作为诊断指标检测肺癌的敏感性为49.5%，特异性为85.0%。其中1个基因甲基化阳性患肺癌的相对危险度为5.28。Tan等^[81]^检测了肺癌患者血浆中*RUNX3*甲基化为55%(11/20)，*p16*为50%(10/20)，*RASSF1A*为30%(6/20)，*CDH1*为20%(4/20)，以这4个基因为标志物检测肺癌阳性率为90%(19/20)。Hsu等^[82]^筛选6个基因组合(*BLU*、*CDH13*、*FHIT*、*p16*、*RARβ*和*RASSF1A*)，其肿瘤和血浆样本甲基化的一致率分别为86%、87%、80%、75%、76%和84%，以其中血浆中检测到两个基因甲基化为诊断标准筛查肺癌的敏感性为73%，特异性为82%。Ostrow等^[83]^选择4个基因组合(*DCC*、*Kif1a*、*NISCH*和*Rarβ*)检测肺癌的敏感性为73%，特异性为71%，结合CT能更好的诊断肺癌。Begum等^[84]^筛选了6个基因组合(*APC*、*CDH1*、*MGMT*、*DCC*、*RASSF1A*和*AIM*)，其中*DCC* 1个基因的检测敏感性为35.5%，特异性为100%，而6个基因的组合检测敏感性增加到75%，而特异性下降到73%。

然而血液中DNA甲基化检测的最大不足之处是缺乏组织特异性，目前检测的这些甲基化的基因标志物不仅在肺癌中有甲基化，在很多其它肿瘤中也有甲基化，尚未发现1个标志物只在肺癌中有甲基化。因此血液中的甲基化检测异常的结果还需要结合特异性更高的影像学检查，以确定肿瘤的位置。目前随着甲基化芯片、焦磷酸测序等技术用于肺癌基因甲基化的研究中，期望能发现肺癌特异性的甲基化标志物。另外，在肺来源的检测介质中检测DNA甲基化有望增加检测的特异性。

### 2.3 痰液中DNA甲基化的检测

痰液含有来自肺和下呼吸道的脱落细胞，具有一定的特异性，痰液中的甲基化检测也有较多报道。Belinsky等^[28]^最早在痰液中检测了*p16*甲基化，在7例肺癌患者中发现3例有*p16*甲基化，而26例对照仅有5例发现*p16*甲基化，有明显差异。随着检测技术的改进和敏感性的提高，Palmisano等^[85]^报道肺癌发生前3年在所有鳞癌患者的痰液中就能检测出*p16*和/或*MGMT*的甲基化。Liu等^[41]^检测了50例肺癌患者，其中痰液中*p16*甲基化率为76%，并发现只有肿瘤组织有*p16*甲基化的肺癌患者痰液中检测到p16甲基化。Wang等^[86]^报道肺癌中*hMLH1*启动子区甲基化阳性率为55.8%，而在痰液中检测的一致率为72%。Olaussen等^[87]^检测发现了95.5%的肺癌患者痰液中细胞学检测阴性，而DNA甲基化检测却有阳性发现。Belinsky等^[88]^检测了肺癌患者和吸烟者痰液中的*p16*、*MGMT*、*RASSF1A*和*DAPK*等7个基因，发现 > 3个基因甲基化阳性肺癌患者是吸烟者的6.2倍。进一步设计病例对照研究^[89]^发现，基因甲基化随着诊断肺癌的时间接近而增加，以*p16*、*MGMT*、*DAPK*、*RASSF1A*、*PAX5b*和*GATA5*这6个基因作为评价指标，其中≥3个基因甲基化阳性肺癌风险增加6.5倍，筛查肺癌的敏感性和特异性达到65%。其它在痰液中评价的基因还有*ASC*/*TMS1*^[90]^和*HOXA9*^[91]^。但是痰液作为检测介质的局限主要来自中央大气道，因此对于鳞癌的诊断价值比较大，而对于主要在肺周围的腺癌则可能不太适合。

### 2.4 支气管灌洗液中DNA甲基化的检测

同痰液类似，支气管灌洗液(bronchoalveolar lavage, BAL)来源于肺的特定肺叶，可能含有肺癌细胞或者DNA可用于肺癌早期诊断。虽然获取支气管灌洗液无创，但需要借助气管镜，对于患者来说还是一项比较痛苦的操作，因此限制了其在大规模筛查中的应用。但是对于怀疑肺癌的高危患者，气管镜是必做的检查，因此对于这部分人群BAL还是容易获得和理想的检查介质。Ahrendt等^[92]^最早检测了BAL中的*p16*甲基化，发现50例患者的肺癌组织中有19例发现*p16*甲基化，这19例患者中11例在BAL中检测到*p16*甲基化。Grote等^[93, 94]^报道在支气管灌洗液中*APC*甲基化诊断肺癌的敏感性达98.5%，而特异性为39%，联合*p16*和*RARβ2*诊断肺癌敏感性69%，特异性达87%。Kim等^[95]^检测了85例NSCLC患者的BAL发现68%的样本至少检出*p16*、*RARβ*、*H-cadherin*和*RASSF1A*其中之一的甲基化。Topaloglu等^[96]^报道了联合检测*APC*、*MGMT*、*RASSF1A*、*p16*等8个基因，在肺癌患者BAL中的阳性率为68%。Schmiemann等^[97]^的一项回顾性病例对照研究中报道247例可疑肺癌患者(确诊89例)的BAL中，联合*APC*、*p16*和*RASSF1A*甲基化检测的诊断敏感性为53%，特异性为99%。

### 2.5 呼出气冷凝液中DNA甲基化的检测

呼出气检测是近年来处于探索阶段的比较新的无创检测方式，在诊断哮喘、慢性阻塞性肺病等肺疾病中已初步体现出应用价值。呼出气冷凝液(exhaled breath condensate, EBC)中除了大部分水蒸气外，还含有脂质、蛋白质和DNA等，可用于肺癌的诊断^[98]^。而目前在EBC中检测DNA甲基化的报道较少，Han等^[99]^检测17例肺癌患者和37例健康对照的EBC中*DAPK*、*RASSF1A*和*PAX5β*启动子区甲基化情况，初步证实了EBC中DNA来源于肺，检测其基因甲基化模式可以区分出肺癌患者和吸烟对照人群。呼出气冷凝液中DNA甲基化检测在肺癌诊断中的价值尚需要更多研究探索。

## 3 问题与展望

尽管近年来DNA甲基化用于早期肺癌诊断受到广泛的关注，对大量的DNA甲基化标志物应用于肺癌诊断的价值也进行了探索和验证，但是目前DNA甲基化分子标志物应用于临床肺癌诊断还存在着一些问题。首先由于采取的检测方式、检测的人群以及病理类型的不同，各个基因在不同报道中的甲基化阳性率有较大差异；其次作为肿瘤标记物，单个基因的阳性率不高，特异性很高的位点极少，达不到用于肺癌早期筛查的标准；另外，用于临床诊断和筛查的标本应该是无创或微创方式得到的介质，如痰液、血液等，而大量的DNA甲基化标志物尚未在这些标本中进行验证。随着研究的不断深入和各种高通量研究手段的应用，更多敏感性和特异性高的DNA甲基化标志物有望被发现并用于肺癌早期诊断试剂盒的开发，成为肺癌早期诊断的有力工具。
